# Euglycemic diabetic ketoacidosis caused by canagliflozin: a case report

**DOI:** 10.1186/s12245-020-0261-8

**Published:** 2020-01-22

**Authors:** Masafumi Fukuda, Masakazu Nabeta, Takanori Muta, Kei Fukami, Osamu Takasu

**Affiliations:** 1 Division of Nephrology, Department of Medicine, Kurume University School of Medicine, 67 Asahi Kurume, Fukuoka, 830-0011 Japan; 2 Advanced Emergency Medical Service Center, Kurume University Hospital, Department of Emergency and Acute Intensive Care Medicine, Kurume University School of Medicine, 67 Asahi, Kurume, Fukuoka, 830-0011 Japan

**Keywords:** Canagliflozin, Sodium glucose cotransporter 2 inhibitors (SGLT2i), Euglycemic diabetic ketoacidosis (eu-DKA)

## Abstract

**Background:**

Diabetic ketoacidosis (DKA) is seen relatively frequently in the emergency department (ED). DKA is characterized by hyperglycemia, acidosis, and ketonemia, and sodium glucose transporter 2 inhibitors (SGLT2i) represent a new diabetes medication that has been associated with euglycemic DKA (eu-DKA).

**Case presentation:**

A 71-year-old female who was being treated for type 2 diabetes with canagliflozin, metformin, and saxagliptin orally presented to the ED for evaluation of reduced oral intake, malaise, nausea, and abdominal pain. Although her blood glucose was not severely elevated (259 mg/dL), there was notable ketoacidosis (pH 6.89; CO_2_, 11.4 mmHg; HCO_3_, 1.9 mEq/L; base excess, − 31.3 mmol/L; 3-hydroxybutyric acid > 10,000 μmol/L) was observed. The uncontrolled acidosis improved following 3 days of continuous renal replacement therapy, but elevated urinary glucose continued for more than 10 days. Ringer’s lactated fluid supplementation was continued for management of polyurea and glucosuria. Urinary glucose turned negative on day 16, and there was improvement in the patient’s overall state; hence, she was discharged on day 18.

**Conclusion:**

Although it is difficult to diagnose eu-DKA because of the absence of substantial blood glucose abnormalities in the ED, there is a need to consider eu-DKA when evaluating acidosis in a patient treated with SGLT2i. Moreover, even after discontinuing the SGLT2i, attention should be given to the possibility of continuing glucosuria. Regular measurements of urinary glucose should be obtained, and the patient should be monitored for dehydration.

## Background

Diabetes is a common disease, and the number of patients is increasing every year. In 2013, 382,000,000 people had diabetes in 219 countries and territories, and it is estimated that the number will rise to 592,000,000 people by 2035 [1]. Diabetic ketoacidosis (DKA) often accompanies diabetes and requires urgent care. DKA is generally defined by hyperglycemia (> 250 mg/dL), acidosis, and ketonemia [2] and is often suspected and diagnosed when notable hyperglycemia is observed. On the other hand, in 1973, Munro et al. proposed a new type of DKA that is not accompanied by hyperglycemia called euglycemic DKA (eu-DKA) [3]. eu-DKA is characterized by a mild increase in blood glucose; thus, DKA cannot be suspected on the basis of a markedly elevated glucose, which could result in delayed diagnosis and a delay in starting treatment [4]. Causes of eu-DKA include gestational diabetes, reduced calorie intake, excessive alcohol consumption, use of cocaine, pancreatitis, and chronic hepatitis [5]. In addition to these factors, there have recently been reports of sodium glucose transporter 2 inhibitors (SGLT2i)—used in the treatment of type 2 diabetes—as a cause [6]. Therefore, it is important for emergency providers to recognize this association. We present a case of eu-DKA in a diabetic patient being treated with SGLT2i who was monitored by blood ketone levels, daily urinary glucose, and urine volume measurements with a favorable outcome. The clinical data were obtained with the patient’s consent for scientific report publication.

## Case presentation

A 71-year-old female who had been undergoing oral treatment for diabetes with canagliflozin 100 mg/day, metformin 1,000 mg/day, and saxagliptin 5 mg/day noticed general malaise a month before her emergency department (ED) visit to Kurume University Hospital Advanced Emergency Medical Service Center. Two weeks before her ED visit, her oral intake decreased because of reduced appetite, but oral medications continued at their initial dose. The day prior to her ED visit, her malaise worsened, and she developed nausea and abdominal pain. She consulted a local doctor where it was discovered that she had notable metabolic acidosis (pH, 6.860; CO_2_, 8 mmHg, HCO_3_, − 1.0 mEq/L; base excess measurement, below sensitivity); hence, she was urgently transferred to our facility. Her vital signs on admission were as follows: body temperature, 35.0 °C; pulse, 118/min; respiratory rate, 28/min; and blood pressure, 111/75 mmHg. The findings on consultation were notable dryness inside the oral cavity and a cold sensation on distal regions of the limbs. Auscultation of the chest revealed no significant findings, but there was mild tenderness in the lower abdominal region. The blood tests at the time of ED evaluation are shown in Table 1. Blood gases demonstrated notable metabolic acidosis (pH, 6.89; CO_2_, 11.4 mmHg; H CO_3_, 1.9 mEq/L; base excess, − 31.3 mmol/L), but the increase in lactic acid was mild at 3.3 mmol/L, and blood sugar was mildly elevated at 259 mg/dL. Although hyperkalemia was observed accompanying the acidosis, no significant kidney function impairment was observed. We considered a diagnosis of lactic acidosis secondary to metformin, but the patient had not recently been exposed to a contrast agent and the lactate was only mildly elevated, making this diagnosis less likely. Since point-of-care urine testing showed strongly positive urinary ketones and glucose, and plasma 3-hydroxybutyric acid (> 10,000 μmol/L) was also markedly high, DKA was suspected even though there was minimal hyperglycemia. As treatment for DKA, an injection of 0.45% sodium chloride was given as an intravenous drip at 800 mL/h. Blood sugar dropped down to 180 mg/dL after using two units of short-acting insulin; hence, insulin was not used continuously. Since hypoglycemia was a concern, an intravenous drip of glucose was started, and around 6 units/day of insulin was used as appropriate while blood sugar was monitored. Notable acidosis continued after starting treatment, and tachypnea and nausea were not tolerable; sodium bicarbonate was given as an intravenous drip. Although conservative treatments were continued, control of the severe ketoacidosis (pH, 7.18; CO_2_, 13.5 mmHg; HCO_3_, 5.0 mEq /L; base excess, − 22.3 mmol/L; 3-hydroxybutyric acid, 9,253 μmol/L) was difficult; thus, from day 2 after hospital admission, continuous renal replacement therapy (CRRT) was started. The acidosis improved because of CRRT over 3 days. The follow-up progress is shown in Table 2. Hyperketonemia reduced notably after a few days, but urinary sugar continued to rise. Osmotic diuresis set in with urinary glucose as the cause and polyurea was observed; thus, fluid replacement was carried out using Ringer’s solution based on the movement of the urinary sugar. Urinary sugar turned negative on day 16 after admission, and as her overall condition was good, she was discharged on day 18 after admission.

**Table 1:** Laboratory data on admission

Biochemical analysis					
Blood gas analysis		Reference range	Biochemistry		Reference range
pH	6.828	7.380–7.460	BUN	18 mg/dL	8–20 mg/dL
PaCO_2_	11.4 mmHg	32.0–46.0 mmHg	Cre	2.02 mg/dL	0.65–1.07 mg/dL
PaO_2_	176.0 mmHg	74.0–109.0 mmHg	Na	143 mEq/L	138–145 mEq/L
HCO_3_^−^	1.9 mmol/L	21.0–29.0 mmol/L	K	4.6 mEq/L	3.6–4.8 mEq/L
BE	− 31.3 mmol/L	− 2.0–2.0 mmol/L	Cl	107 mEq/L	101–108 mEq/L
Lactate	3.3 mmol/L	0.44–2.13 mmol/L	CRP	5.78 mg/dL	≤ 0.14 mg/dL
Urinalysis			HbA1c	6.7%	4.9–6.0%
Glucose	4 +		3-hydroxybutyric acid	> 10,000 μmol/L	< 74 μmol/L
Ketone	3+				

Abbreviations: *pH* power of hydrogen, *PaO*_*2*_ partial pressure of arterial oxygen, *PaCO*_*2*_ partial pressure of arterial carbon dioxide, *HCO*_*3*_ hydrogen carbonate, *BE* base excess, *BUN* blood urea nitrogen, *Cre* creatinine, *Na* sodium, *K* potassium, *Cl* chloride, *CRP* C-reactive protein, *HbA1c* glycated hemoglobin

**Table 2:** Initial and follow-up laboratory findings

Parameter	Initial	After 3 days	After 6 days	After 9 days	After 12 days	After 16 days
Arterial pH [7.380–7.460 mmHg]	6.828	7.406	7.484	7.456		
Serum bicabonate [21.0–29.0 mmol/L]	1.9	17.4	27.0	26.1		
Serum 3-hydroxybutyric acid [< 74 μmol/L]	> 100,000	2695	782	333	156	79
Serum lactate [0.44–2.13 mmol/L]	3.3	0.7	0.5	0.5		
Urine volume [mL/day]	6400	5380	3750	2010	1950	890
Urine sugar [< 85 mg/day]	73,107	92,966	71,636	44,700	19,390	0

Abbreviation: *pH* Power of hydrogen

## Discussion and conclusions

SGLT2i became available in 2013 and are the latest oral hypoglycemic agent for type 2 diabetes [7]. They reduce blood sugar by inhibiting the reabsorption of glucose and inducing the excretion of glucose in the urine [8]. Moreover, SGLT2i improve the functionality of pancreatic β cells, promote weight loss, reduce blood pressure, and have been shown to reduce cardiovascular deaths and mortality rates caused by other factors [9]. Hence, currently, SGLT2i are recommended as the second-line medication after metformin [10]. In general, complications accompanying the use of SGLT2i are urinary tract infection and genital infections [9]. Although eu-DKA has been reported to accompany the use of SGLT2i, it is not well recognized. Two points are emphasized in this thesis. The first point is that eu-DKA is possible if SGLT2i are used. The second point is that although hyperketonemia reduces relatively quickly, urinary sugar (with or without polyurea) continues for over a week. Canagliflozin used in this case was SGLT2i, which was first available in 2013 [11]. Previous research relating to type 2 diabetes showed that the rate of onset of DKA among patients treated with canagliflozin is more than twice the DKA onset rate among patients treated with oral hypotensive agents other than canagliflozin [12]. Although canagliflozin may cause DKA, it does not commonly result in eu-DKA. Canagliflozin increases urinary glucose excretion in a dose-dependent manner, and the half-life at a 100 mg dose (as per our case) is estimated to be 10.6 h [13]. Therefore, the effect of SGLT2i is thought to disappear 2–3 days after discontinuing the use of canagliflozin [14]. However, in this case, a high level of urinary sugars was observed for 12 days. There is a previous case report of a similar eu-DKA that occurred as a result of canagliflozin, for which the urine glucose level continued to be > 500 mg/dL for 9 days with no hyperglycemia observed [15]; this suggests that eu-DKA due to canagliflozin leads to extended increases in urinary sugar. Canagliflozin is metabolized by the uridine diphosphate glycosyltransferase (UGT) enzyme [13], and it is possible that UGT polymorphisms contribute to the long-term effect of the drug [16]; however, proving the mechanism underlying the role of SGLT2i in the onset of eu-DKA was difficult in this case. Although further research is required to determine the mechanism, significant urinary sugar due to canagliflozin can trigger the induction of osmotic diuresis. Treatment should be given, and dehydration levels in the patient should be monitored while urinary sugars are still being passed.

For this case, CRRT was performed in respect of ketoacidosis, which was difficult to control. There have been reports in the past of CRRT being performed for 2 days in respect of persistent ketoacidosis, with good results [17]. In this case, CRRT was given for 3 days, without any complications. There was no re-deterioration of the ketoacidosis after the completion of CRRT; thus, short-term CRRT should be considered as a treatment option for acute-phase ketoacidosis when correction proves to be difficult.

eu-DKA is defined when blood sugar is < 300 mg/dL, and plasma bicarbonate is < 10 mEq/L [3], and since notable hyperglycemia is not seen, it is difficult to diagnose suspected ketoacidosis in the ED. In order to suspect it, there is a need to be aware of the possibility of eu-DKA with “normal blood sugar levels” due to SGLT2i. Additionally, in patients who have been limiting their carbohydrate intake, there is greater possibility of eu-DKA being caused by the use of SGLT2i [18]. If someone using SGLT2i is seen to have reduced oral intake and shows symptoms such as general malaise, abdominal pain, polypnea, and drowsiness, then eu-DKA is one potential identifiable cause. It should be noted that cases of eu-DKA accompanying SGLT2i use may experience elevated urinary glucose for around 10 days, even after stopping SGLT2i and with no evidence of hyperglycemia. Additionally, there is a need to measure urinary sugar regularly and monitor dehydration levels, with urinary glucose as a marker.

## Data Availability

The datasets used the current study are available from the corresponding author on reasonable request.

## Abbreviations

CRRT: Continuous renal replacement therapy
DKA: Diabetic ketoacidosis
ED: Emergency department
eu-DKA: Euglycemic diabetic ketoacidosis
SGLT2i: Sodium glucose transporter 2 inhibitors
UGT: Uridine diphosphate glycosyltransferase
